# Direct from the Seed: An Atomic-Resolution Protein Structure by Ab Initio MicroED

**DOI:** 10.1101/2025.07.03.663097

**Published:** 2025-07-05

**Authors:** Purna Chandra Rao Vasireddy, Timothy Low-Beer, Katherine A. Spoth, Devrim Acehan, Matthew R. Crawley, Michael W. Martynowycz

**Affiliations:** 1 Department of Structural Biology, Jacobs School of Medicine and Biomedical Sciences, University at Buffalo, The State University of New York, Buffalo, NY 14203; 2 UB Hauptman-Woodward Institute, University at Buffalo, The State University of New York, Buffalo, NY 14203; 3 Department of Chemistry, University at Buffalo, The State University of New York, Buffalo, NY 14260

**Keywords:** Microcrystal electron diffraction, Cryo-EM, Phasing, Atomic Resolution, Natural Product

## Abstract

While purifying the seed protein crambin, we discovered that needles of pure protein nanocrystals formed spontaneously during the drying of a simple ethanolic purification drop. Contrary to traditional crystallography, these needles diffracted poorly using X-rays yet proved to be exceptionally well-suited for microcrystal electron diffraction (MicroED). By merging data from 58 such nanocrystals, we obtained diffraction to 0.85 Å resolution with an overall correlation coefficient of over 99% and solved the structure *ab initio* using a five-residue helical fragment to initiate density modification. The resulting map was of exceptional quality, enabling fully automated model building and resolving individual hydrogen atoms. This work represents the highest-resolution protein structure (0.85 Å) determined from spontaneously formed protein nanocrystals and is the first *ab initio* structure of crambin solved by electron diffraction. Our workflow demonstrates that complex biological matrices can be mined directly for sub-ångström protein structures, establishing a practical and scalable pipeline from raw biomass to atomic-level models of previously intractable targets.

## Introduction

Crystallography, the cornerstone method for determining protein structures, has traditionally been limited by crystal size. X-ray crystallography, the traditional gold standard, demands relatively large, well-ordered crystals - typically tens to hundreds of micrometers across. Yet many proteins naturally form only extremely tiny crystals, far smaller than this threshold, leaving a significant fraction of important biological molecules structurally inaccessible. Single-particle cryo-electron microscopy (cryo-EM), another powerful approach, struggles to achieve high-resolution structures of proteins less than 100 kDa.^1^ This limits its utility for many biologically important molecules, e.g. the median size of a human protein is only about 40 kDa.^2^

Microcrystal electron diffraction (MicroED) has emerged as a transformative approach to overcome these limitations.^3^ Instead of X-rays, MicroED uses electrons, which interact far more strongly with matter.^4^ This powerful interaction allows high-resolution structure determination from crystals thousands of times smaller by volume than those required for traditional X-ray methods. Crystals invisible to the naked eye,^5^ and often too delicate or too small for standard crystallography, can now yield atomic-resolution structures. This opens the door to streamlined ‘powder-to-structure’ workflows, where complex sample optimization is replaced by direct analysis of nanocrystalline material.^6,7^

Despite its promise, phasing novel protein structures by MicroED remains heavily reliant on pre-existing knowledge. Molecular replacement (MR), first demonstrated with lysozyme in 2013–2014,^8,9^ is the workhorse method and has delivered important structures like R2lox,^10^ MyD88,^11^ and the human lens membrane protein MP20.^12^ The arrival of AlphaFold2^13^ has further expanded the reach of MR. However, this reliance on homologous models creates a fundamental limitation: it generates phase bias and leaves proteins with novel folds entirely inaccessible. Establishing reliable ab initio phasing methods that remove this dependence is essential. While this has been routine for small-molecule electron crystallography for years,^6,14–16^ progress for proteins has been incremental, moving from direct methods on small peptides,^17^ to fragment-based searches,^18^ and recently to density-modification-based bootstrapping from minimal ideal fragments.^19^ Our work confronts this challenge directly, providing a robust, atomic-resolution benchmark for this latter approach, of which there are still very few examples.

In this study, we bridge this methodological gap by using the classic benchmark protein crambin.^20^ For decades, crambin has served as a proving ground for the most advanced crystallographic methods. Its 1981 structure by Hendrickson and Teeter was a landmark in *de novo* phasing using sulfur anomalous scattering,^21^ and it was later the first protein solved by Hauptman and Weeks’ purely mathematical direct methods, a triumph developed at our home institutions. In subsequent years, synchrotron and neutron^22^ sources pushed its resolution to an extraordinary 0.48 and 0.54 Å,^23,24^ making it a key model for studying ultra-high-resolution features.

Despite this storied history in X-ray and neutron science, a complete *de novo* investigation of crambin by MicroED has remained unexplored. While a 1.07 Å MicroED structure solved by molecular replacement has been briefly described in a technical note (Rigaku Application note PX031),^25^ the full potential of using electrons to solve this benchmark protein from first principles has not been realized. We discovered that a simple ethanolic extraction from ground seeds spontaneously yields an abundant supply of nanocrystals ideal for such a test. Therefore, we set an ambitious goal: to determine if data from these naturally formed nanocrystals were of sufficient quality to not only break the sub-ångström barrier but to solve the structure entirely *ab initio* from a minimal, non-homologous five-residue helical fragment.^26^

Successful *ab initio* structure determination would represent the first electron-based atomic-resolution structure of this pivotal protein, validating a streamlined pipeline from raw biological material directly to detailed structural models. This result not only provides an essential benchmark for advancing electron crystallography but also demonstrates a practical, scalable approach capable of unlocking structural information from previously underexplored biological targets, such as naturally occurring macromolecular products.

## Results and Discussion

### A Tale of Two Crystals: Swapping Fortunes in X-ray and Electron Diffraction.

**Figure 1.**
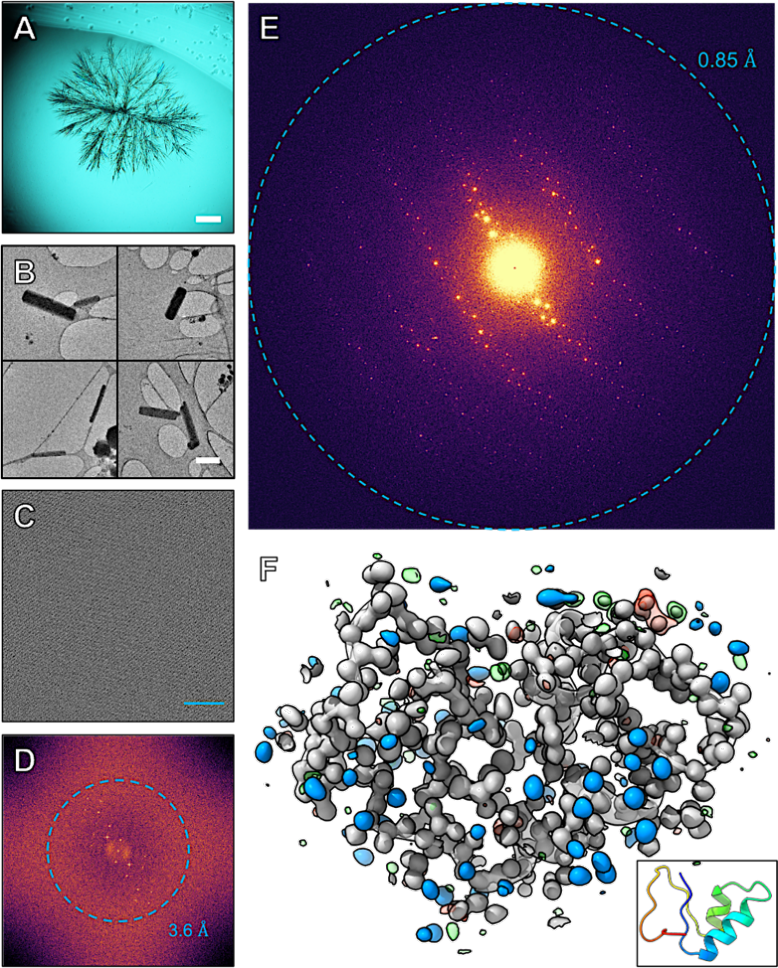
Characterization of crambin microcrystals. (**A**) Crambin protein crystals crashed out in a drop on a cover slide. Scale bar 125 μm. (**B**) Collection of four TEM images at 4300 × magnification showing small rod-shaped crystals of crambin. Scale bar 1 μm. (**C**) High resolution TEM image of the crystal lattice of a typical crambin crystal in the TEM. Scale bar 200 Å. (**D**) Fourier transform of panel C showing lattice order out to 3.6 Å. (**E**) 1° of MicroED data showing diffraction to 0.85 Å resolution. (**F**) The final refined model of crambin as a transparent cartoon. The 2F_o_-F_c_ map is in gray and contoured to 1.5 σ. The blue densities are the same map segmented around the modelled water residues. The F_o_-F_c_ map with a threshold at +3 σ is in green and with a threshold of −3 σ is shown in red. A cartoon depiction of the final model is inset in the same orientation.

Our investigation began with a serendipitous discovery. During a simple ethanolic extraction of crushed *Crambe abyssinica* seeds (Supplementary Figure 1), we found that a 1 μL droplet of the protein-rich solution nucleated a dense shower of sub-micron, needle-shaped crystals within seconds as the solvent evaporated (Figure 1A). No crystallization screen is required. The process is rapid and spontaneous. This shortens the entire pipeline from raw seed to refined atomic model. The process can be completed in a week or less. These needles proved unsuitable for X-ray diffraction. A single 14 μm needle diffracted poorly, only reaching 1.6 Å resolution and suffered from rapid radiation decay (Supplementary Figure 2, Supplementary Table 1). In contrast, they proved ideal for MicroED. When loaded into the TEM, we immediately noticed many tiny rod-shaped crystals on the grid (Figure 1B). High resolution imaging of these crystals showed clear lattice lines (Figure 1C) that contained information to 3.6 Å by analysis of the Fourier transform of these images (Figure 1D). These crystals consistently diffracted electrons to high resolution (Figure 1E).

We also grew large, block-like crystals using traditional vapor diffusion (Supplementary Figure 3). These crystals exhibited the opposite behavior: they produced excellent X-ray diffraction to beyond 0.8 Å but were initially considered a secondary choice for MicroED due to their thickness. Initially, we mechanically crushed these high-quality blocks to obtain fragments small enough for MicroED. The rationale was twofold: first, we hoped to leverage their superior internal order observed with X-rays; second, we anticipated that randomly oriented fragments would mitigate the severe preferred orientation expected from the needle morphology, thereby yielding a more complete dataset. However, this approach failed. The crushed fragments produced markedly inferior electron diffraction data compared to the spontaneously formed needles (Supplementary Figure 4).

This surprising inversion of crystal quality between the two diffraction methods became a central finding of our work. The gentle, in-solution self-assembly of the nanocrystals preserves a higher degree of internal order for electrons, while the block-like crystals that excel with X-rays are damaged by the mechanical stress of fragmentation. This led us to a critical decision: instead of pursuing randomly oriented but damaged crystal fragments, we would tackle the preferred orientation problem of the spontaneously formed needles directly using a serial crystallography approach. By merging data from dozens of crystals, we aimed to overcome geometric data loss inherent to a single orientation and build a complete, high-redundancy dataset.

### Achieving an Atomic-Resolution MicroED Dataset by Overcoming Anisotropy.

Having established the superiority of the spontaneously formed nanocrystals for MicroED experiments, we proceeded with data collection. The primary technical hurdle was, as anticipated, the severe preferred orientation of the needles on the grid.^27^ This morphology introduces a fundamental challenge for MicroED: diffraction quality is highest at low tilt angles where the electron beam passes through the thinnest dimension of the crystal but degrades at high tilts as the effective crystal thickness increases,^28^ compounding multiple scattering and absorption effects. This inherent tilt-dependent resolution decay is a major source of anisotropy in data from plate- or needle-like crystals.^10^

To obtain a high-quality dataset, we merged data from 58 individual nanocrystals using a serial MicroED strategy. To maximize data completeness and mitigate the “missing wedge” effect inherent to single-crystal collection, we collected a 30° or 60° data wedge from each crystal. The starting angle for each wedge was intentionally varied, ensuring that data from different crystals sampled unique regions of the full goniometer tilt range. Even with this comprehensive collection strategy, the merged dataset exhibited the expected anisotropy. A standard spherical resolution cutoff would therefore be suboptimal, either conservatively low, discarding valuable high-resolution data, or too high, including noise from the poorly ordered directions.

To produce the most physically meaningful and high-quality dataset, we chose to explicitly address this anisotropy. We processed the integrated intensities with the STARANISO server,^29^ which determines the direction-dependent resolution limits and applies a corresponding elliptical truncation. This process correctly removes the weak, high-resolution impaired reflections from the poorly diffracting directions while preserving the strong, high-resolution data from the well-ordered directions. While modern phasing programs like Phaser are robust against some noise, providing them with the cleanest possible dataset maximizes the chances of success in a challenging *ab initio* case. Similarly, refinement benefits from a dataset where resolution limits are physically justified.

The final, anisotropy-corrected dataset extended to an overall resolution of 0.85 Å and exhibited high internal consistency and an effective (ellipsoidal) completeness of 98.6% (Table 1). The high data multiplicity of 42.67, achieved by merging data from 58 nanocrystals, was a critical factor in producing the exceptional signal-to-noise ratio within this well-defined resolution ellipsoid. The diffraction limit is defined by an elliptical cutoff with resolutions of 0.84 Å, 0.85 Å, and 1.49 Å. As recent work by Xu et al. has shown,^30^ high multiplicity can significantly improve the overall quality of merged MicroED datasets, and our result provides a powerful validation of this strategy. Analysis of the data showed no evidence of specific radiation damage.^31^ No significant difference map peaks appeared at susceptible sites such as disulfide bonds or acidic residues.

### Ab Initio Phasing and Automated Structure Completion.

**Figure 2.**
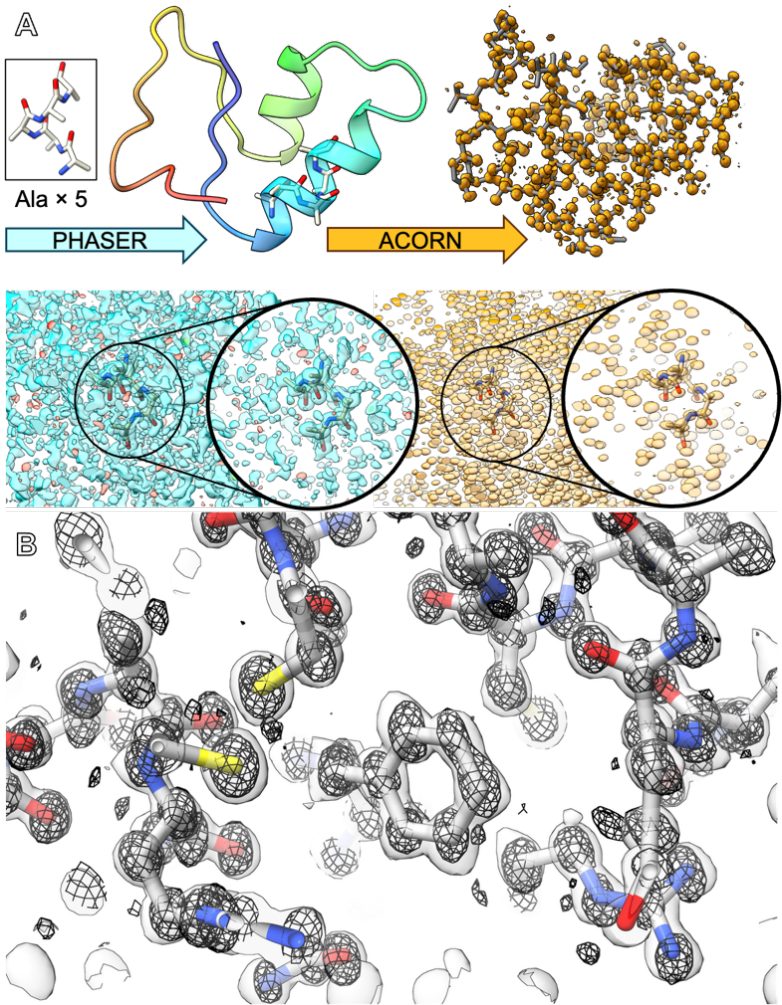
*Ab initio* structure solution of crambin at atomic resolution by MicroED. (**A**) Pipeline showing how the structure was phased. An ideal helical fragment consisting of 5 alanine residues (**top left, inset**) was placed in the unit cell by PHASER. The location of the placement relative to the final structure is shown in the **A, top left**. The resulting map could not be understood or built into outside of the placed fragment (**A, bottom, blue density**). The placed fragment was used to generate initial phases for phase improvement by the density modification program ACORN, which returns a fully interpretable map (**A, right, orange densities**). This map was used to automatically build the entire protein using BUCCANEER (**A, top right**). (**B**) The final refined model and 2F_o_-F_c_ map (**B, surface**) are shown with the original |E| map from density modification, demonstrating the atomic positions (**B, mesh**).

The central achievement of this work was to solve the structure without a homologous model. Our workflow, conceptually similar to the fragment-based Fragon^32^ pipeline developed for X-ray data, began with an attempt at molecular replacement in PHASER^33^ using a generic five-residue poly-alanine helix. This yielded a statistically marginal solution (TFZ = 7.2) with an uninterpretable potential map (Figure 2A); however, these initial phases were sufficient to bootstrap the dynamic density modification algorithm in ACORN.^34^ This fragment-based phasing approach is similar to the strategies recently used to solve the structures of lysozyme and Proteinase K by MicroED,^19^ confirming its power for solving *ab initio* protein structures from high-quality electron diffraction data. The overall concept is also similar to the ARCIMBOLDO^35^ and SHELXE^36^ based approaches. This approach solved the phase ambiguity, producing an exceptionally clear map in which the entire polypeptide chain was visible (Figure 2A). The quality of this *ab initio* map was high enough for BUCCANEER^37^ to automatically build 100% of the crambin model in a single pass. The final structure was refined against the twinned, anisotropy-corrected data to R_work_ / R_free_ values of 15.4% / 17.2%, yielding a model with excellent stereochemistry (Table 1). The resulting 2F_o_-F_c_ map resolves all non-hydrogen atoms and many hydrogen atoms, confirming the genuine atomic resolution of the structure (Figure 1F).

Crambin extracted from seeds contains two natural isoforms known as PL and SI, differing at residues 22 and 25.^38^ Our workflow did not separate these variants; however, the exceptional quality of our final map allowed us to resolve this heterogeneity directly. Refinement of both isoforms with partial occupancies converged to a stable, near 50:50 ratio and, critically, eliminated all significant positive or negative peaks in the difference maps at the sites of variation, confirming the accuracy of the mixture model.

### Forging the Next Link in a Benchmark’s Legacy.

By solving the first *ab initio* electron structure of crambin, our work forges a direct link between the foundational phasing breakthroughs of the 20^th^ century and the new frontier of atomic-resolution electron crystallography. The original solutions of crambin by Hendrickson, Teeter, Hauptman, and Weeks were not merely structural triumphs; they were powerful proofs of principle that validated new phasing methodologies for the entire field.^21,39^ This role as a methodological testbed has continued, including its use as a target for total chemical synthesis to validate protein ligation and folding strategies.^40^ We believe our result serves the same purpose for the modern era of MicroED.

We have shown that a minimal, non-homologous fragment is sufficient to phase atomic-resolution data from nanocrystals obtained without deliberate crystallization. This approach provides crystals of significantly higher quality than those reported from chemically synthesized material.^40^ This achievement transforms crambin from being solely a benchmark for X-ray^24^ and neutron^22,41^ methods into a vital, cross-platform standard. The availability of our 0.85 Å electrostatic potential map alongside the classic electron-density and neutron-scattering-length maps now provides an invaluable resource for calibrating and developing the next generation of electron-specific refinement tools, especially those designed to tackle the grand challenges of dynamical scattering^42^ and charge density analysis.^43^

We have determined an atomic-resolution electron diffraction structure of crambin, at 0.85 Å. The structure was solved *ab initio* using nanocrystals that formed spontaneously, without deliberate crystallization. Although our fragment-based approach differs from sulfur anomalous dispersion or traditional direct methods, it shares their underlying principle: starting from minimal initial information to build a complete structure.

The resulting electrostatic potential map shows precisely how electrons scatter off the Coulomb potential of every atom.^44^ Unlike X-ray maps, which highlight electron clouds, or neutron maps, which locate atomic nuclei,^45^ MicroED maps directly show the electrostatic potential felt by incoming electrons. This potential is sensitive to both the number of electrons and the atom’s net charge. As a result, map peaks for negatively charged atoms appear less intense than expected based on atomic number alone. Meanwhile, even individual hydrogen atoms appear clearly as positive peaks,^46^ rather than being invisible or merely inferred.

Since our map provides a complementary chemical picture of crambin to those available through X-ray or neutron techniques,^47^ crambin once again serves as a benchmark, this time as a gold-standard reference for future electron-specific methods, such as modeling electron scattering, analyzing charge density, and accurately visualizing hydrogen bonds.^46^ In this way, our work connects past breakthroughs in solving the crystallographic phase problem with today’s emerging field of atomic-resolution electron crystallography.

## Conclusions

In this work, we have demonstrated a complete and streamlined pipeline for the *ab initio* structure determination of a protein to atomic resolution using MicroED. We have shown that sub-micron protein needles, formed spontaneously during a simple ethanolic extraction, can be carried directly from crude plant material to a fully refined 0.85 Å structure without the need for extensive crystallization trials or a homologous search model. This low-tech crystallization, high-impact workflow potentially turns raw biological matter into ready-made nanocrystal libraries, enabling structural analysis of historically intractable proteins.

Our findings significantly lower the barrier for obtaining high-resolution structures, particularly for proteins that crystallize only as sub-micron needles or lack known structural homologues. The high redundancy of our merged dataset not only contributed to the quality of the final model, as previously suggested by others,^30^ but also points toward future data collection strategies. We believe that future experiments employing serial electron crystallography^48^ by scanning the beam or the stage with extremely short exposures could further improve data quality by eliminating stage rotation artifacts entirely and vastly increase multiplicity.

The exceptional quality of this crambin dataset provides an ideal benchmark for future electron-based refinement methods. Notably, our R_work_ / R_free_ values (15.4%/17.2%) are among the lowest reported for a protein structure by MicroED, and the data quality metrics (R_pim_ = 3.9%) are consistent with largely kinematic scattering. This is likely due to the use of extremely thin nanocrystals (<100 nm), a highly parallel electron beam, and the use of highly redundant data, which likely minimize any dynamical effects.^28^ Nevertheless, no protein structure has yet been refined with a full dynamical model. Our 0.85 Å data, with its high redundancy and resolution, represents a perfect testbed for developing and validating such tools, which will be essential for pushing the boundaries of accuracy.

The robustness of this system makes it an ideal platform for systematically studying the effects of different solvents, cryoprotectants, or ligands on protein structure at atomic resolution. Furthermore, this high-quality electron-based model, when combined with existing ultra-high-resolution X-ray and neutron data, creates a unique opportunity for joint refinement,^49^ which could provide unparalleled insight into chemical bonding and protonation states. By incorporating energy filtration,^50,51^ faster detectors,^52^ and advanced sample preparation techniques like ion-beam milling,^53,54^ the stage is now set to explore hydrogen bonding networks and charge-density distributions, extending true atomic-resolution structural biology to an ever-wider range of challenging biological systems.

## Materials and Methods

### Protein Isolation and Nanocrystal Formation.

Crambin was extracted from 230 g of manually de-husked and then crushed *Crambe abyssinica* seeds following a modified protocol.^55,56^ The ground kernels were first defatted with hexanes and then subjected to three rounds of extraction with 80% acetone in water (%v/v). After evaporation of the acetone under reduced pressure, the aqueous solution was stored at 4 °C for 18 hours. This solution yielded a pale-yellow solid pellet upon centrifugation, which was redissolved in a 70% ethanol-water mixture (%v/v). While attempts to grow large single crystals by slow cooling or vapor diffusion from this solution were unsuccessful, we made a key discovery during routine sample inspection. When a 1 μL droplet of the ethanolic protein solution was allowed to evaporate on a microscope slide, it rapidly nucleated a dense shower of sub-micron, needle-shaped crystals. These spontaneously formed nanocrystals, unsuitable for X-ray diffraction, became the source material for all subsequent MicroED experiments. The full detailed extraction protocol is available in the Supplementary Methods.

### MicroED Grid Preparation and Data Collection.

Holey-carbon copper grids (Quantifoil Cu 300 R1.2/1.3) or lacey carbon grids (Cu 200, Electron Microscopy Sciences) were glow-discharged (15 mA, 30s). The grids were transferred to either a Leica GP2 plunge freezer or Thermo-Fisher Vitrobot equilibrated to 4 °C and 95% relative humidity. A 3 μL aliquot of the crystalline slurry was applied, blotted for approximately 30s, and plunge-frozen in liquid ethane. MicroED data were recorded on a Glacios TEM (200 kV, λ = 0.0251 Å) equipped with a Falcon 4 detector. Two sets of data were collected using SerialEM:^57,58^Linear-mode datasets with wedges 0.2°/frame and 1.6 e^−^ Å^−2^ total exposure. Counting-mode datasets were collected with 0.1°/frame and 0.8 e^−^ Å^−2^ total exposure. We did this in the hopes the low-resolution reflections in linear mode would be less prone to coincidence loss, and the high-resolution reflections in counting mode would be more accurately recorded. The total exposure per crystal that was used for MicroED data collection was kept below 2 e^−^ Å^−2^. In both schemes the starting angle of each wedge was incremented stepwise so that the combined datasets sample a stage tilt range of +/− 60°. Counting mode and linear mode datasets were collected on different crystals.

### Data processing and Refinement.

Raw movie frames were converted to the miniCBF format using a custom Python pipeline that performed 2×2 binning and a two-pass, smoothed beam-center correction (**see** Supplementary Methods **for full details**). The corrected images were processed with XDS (BUILD 20230630) and scaled with XSCALE. The final merged dataset from 58 crystals was processed by the STARANISO server to correct for anisotropy. Phasing was performed using a combination of Phaser and ACORN, followed by automated model building in BUCCANEER. The final structure was refined using REFMAC5 with explicit modeling of a 10.0% twin fraction. Final data collection and refinement statistics are provided in Table 1.

## Supplementary Material

Supplement 1

Supplement 2

3

## Figures and Tables

**Table 1.** MicroED structure of crambin

Data collection	
No. of crystals	58
Space group	*P* 2_1_
Cell Dimensions	
(a, b, c) (Å)	41.490, 18.790, 22.520
(α, β, γ) (°)	90.00, 90.84, 90.00
Resolution (Å)^1^	22.58 – 0.85 (0.87 – 0.85)
R_pim_ (%)	3.9 (48.2)
< I / σ I >	12.118 (1.00)
Completeness (%)	98.6 (83.6)^1^
Redundancy (#)	42.67 (8.60)
Refinement	
Resolution (Å)	22.58 – 0.85 (0.872 −0.85)
No. Unique Reflections (#)	22,022 (619)
R_work_ / R_free_ (%)	15.4 / 17.2
No. Atoms / B-factors	426 / 6.68
Protein (# / Å^2^)	354 / 4.60
Water (# / Å^2^)	42 / 16.89
RMS deviations	
Bond lengths (Å)	0.021
Bond Angles (°)	1.96
Clash score	2.92

1 Statistics after elliptical truncation and scaling with the STARANISO server.

## Data Availability

Coordinates and structure factors for crambin determined by MicroED are deposited in the PDB and maps from this work are available in the EMDB. All raw MicroED data in MRC format and associated metadata for this work have been uploaded to Zenodo. Data conversion scripts and code used in this paper are available from the PI upon request and from our GitHub page.
