# Supplementary material for: Direct from the Seed: An Atomic-Resolution Protein Structure by Ab Initio MicroED
Source: bioRxiv. 2025 Jul 5:2025.07.03.663097. Preprint. [Version 1] doi: 10.1101/2025.07.03.663097 (PMC12236495; doi:10.1101/2025.07.03.663097)
Supplement: 3 [file NIHPP2025.07.03.663097V1-supplement-3.pdf]

bioRxiv preprint doi: https://doi.org/10.1101/2025.07.03.663097; this version posted July 5, 2025. The copyright holder for this preprint (which was not certified by peer review) is the author/funder, who has granted bioRxiv a license to display the preprint in perpetuity. It is made available under aCC-BY 4.0 International license.

Supplementary Information for:

Atomic-Resolution Protein Structure Directly from Raw Material by Ab Initio MicroED

Purna Chandra Rao Vasireddy,[1] Timothy Low-Beer,[1] Katherine A. Spoth,[2]
Devrim Acehan,[1,2] Matthew R. Crawley,[3] Michael W. Martynowycz[1,2, #]

[1]Department of Structural Biology, Jacobs School of Medicine and Biomedical Sciences, University at Buffalo, The State University of New York, Buffalo, NY 14203
[2]UB Hauptman-Woodward Institute, University at Buffalo, The State University of New York, Buffalo, NY 14203
[3]Department of Chemistry, University at Buffalo, The State University of New York, Buffalo, NY 14260
[#]Correspondence: mmartyno@buffalo.edu

List of Supplementary Items

1. **Supplementary Figure 1**
2. **Supplementary Figure 2**
3. **Supplementary Figure 3**
4. **Supplementary Figure 4**
5. **Supplementary Table 1**
6. **Supplementary Table 2**
7. **Supplementary Methods**
    a. **Materials**
    b. **Protein isolation and crystallization**
    c. **X-ray crystallography control experiments**
    d. **MicroED grid preparation**
    e. **TEM lattice imaging**
    f. **MicroED data collection**
    g. **Conversion of MRC movies to miniCBF format**
    h. **Data Integration, Scaling, and Anisotropy Correction**
    i. **Phasing, Model Building, and Refinement**
    j. **Figure generation**

8. Supplementary Video 1

9. Supplementary Video 2

bioRxiv preprint doi: https://doi.org/10.1101/2025.07.03.663097; this version posted July 5, 2025. The copyright holder for this preprint (which was not certified by peer review) is the author/funder, who has granted bioRxiv a license to display the preprint in perpetuity. It is made available under aCC-BY 4.0 International license.

1. Supplementary Figure 1

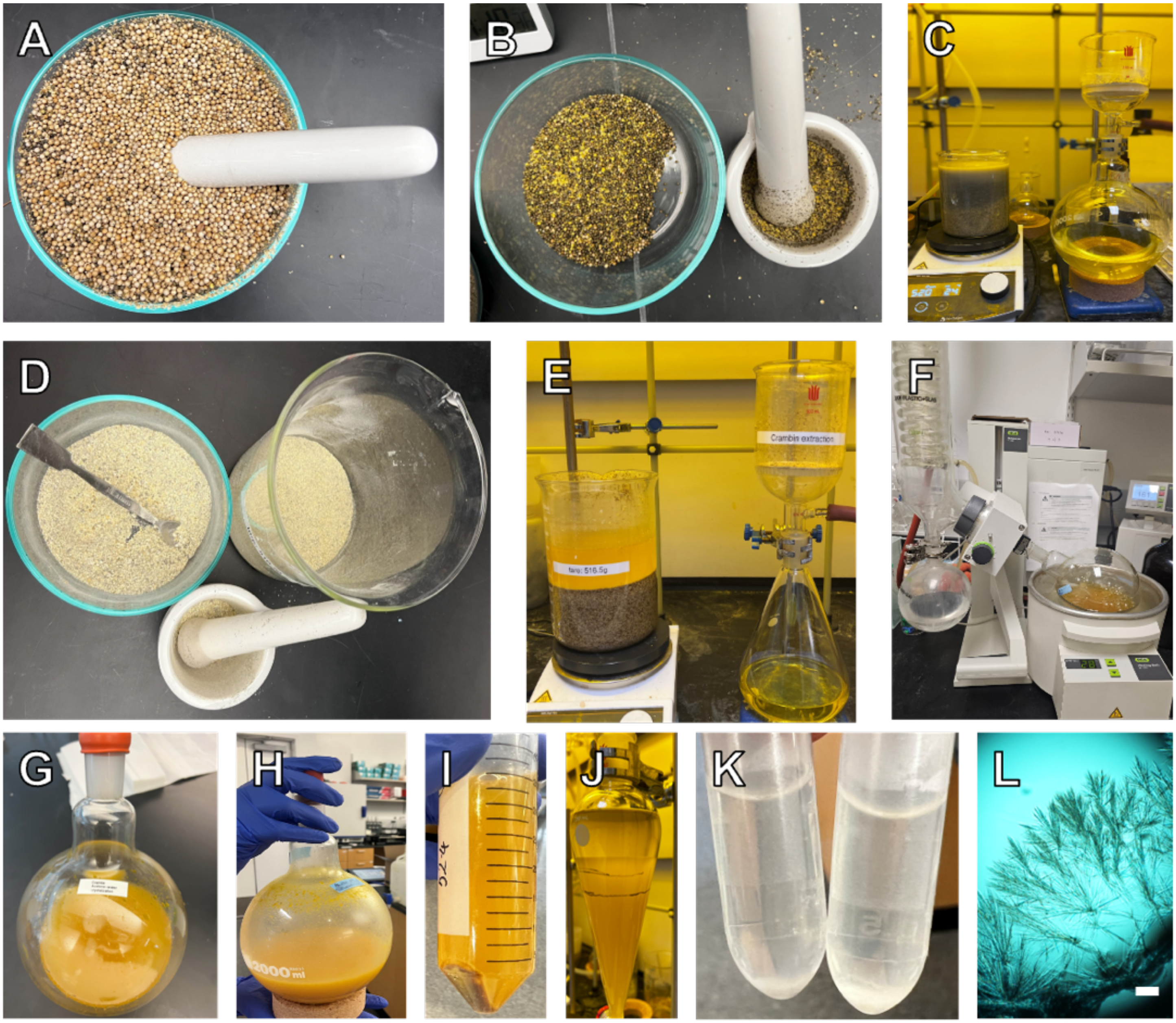

Supplementary Figure 1. The Crambin Extraction Process. (**A**) Husking crambin seeds. (**B**) Ground seeds prior to defatting. (**C**) Defatting by mixing with hexanes and filtering. (**D**) Ground seed meal prior to crambin extraction. (**E**) Crambin extraction using 80% acetone-water by mixing and filtering. (**F**) Acetone removal under reduced pressure and <40°C. (**G**) Solution after acetone removal. (**H**) solution after 18h at 4°C, (**I**) centrifuged solution. (**J**) hexane washings to crude crambin pellet dissolved in 70% EtOH-water. (**K**) crystals obtained by slow evaporation. (**L**) microcrystals seen under light microscope. Scale bar 125 μm.

bioRxiv preprint doi: https://doi.org/10.1101/2025.07.03.663097; this version posted July 5, 2025. The copyright holder for this preprint (which was not certified by peer review) is the author/funder, who has granted bioRxiv a license to display the preprint in perpetuity. It is made available under aCC-BY 4.0 International license.

2. Supplementary Figure 2.

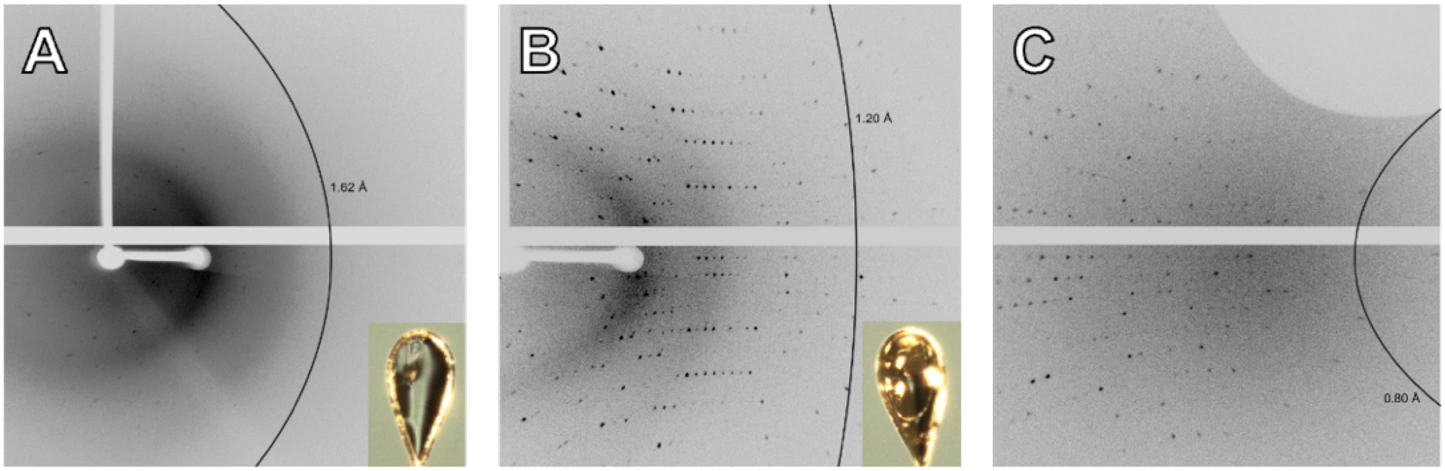

Supplementary Figure 2. Comparison of needle shaped crystals grown by evaporation to block shaped crystals grown by vapor diffusion by single crystal X-ray crystallography. (**A**) A single frame (0.5° width) of a needle-like crystal of crambin (see inset) with a 120 second exposure time and resolution ring at 1.62 Å. (**B**) A single frame (0.5° width) of a block-like crystal of crambin (see inset) with a 10 second exposure time and resolution ring at 1.20 Å. (**C**) The same crystal from panel B with a (0.5° width) with a 35 second exposure time showing diffraction beyond the resolution ring at 0.80 Å.

bioRxiv preprint doi: https://doi.org/10.1101/2025.07.03.663097; this version posted July 5, 2025. The copyright holder for this preprint (which was not certified by peer review) is the author/funder, who has granted bioRxiv a license to display the preprint in perpetuity. It is made available under aCC-BY 4.0 International license.

3. Supplementary Figure 3.

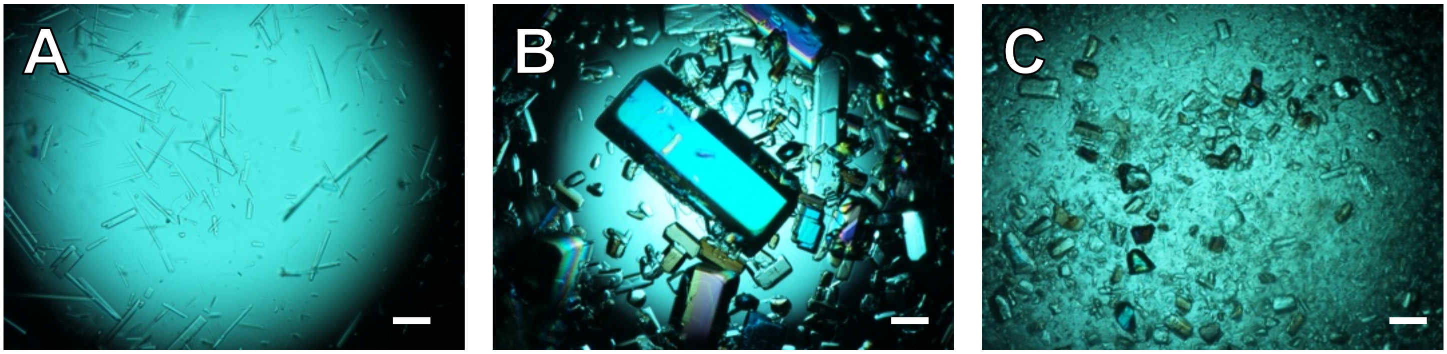

Supplementary Figure 3. (**A**) Crashed out needles of crambin formed by slow evaporation. (**B**) large block shaped crystals of crambin with sizes up to 1 mm across. (**C**) Crystals from panel B after being mechanically crushed. All scale bars 125 µm.

4. Supplementary Figure 4.

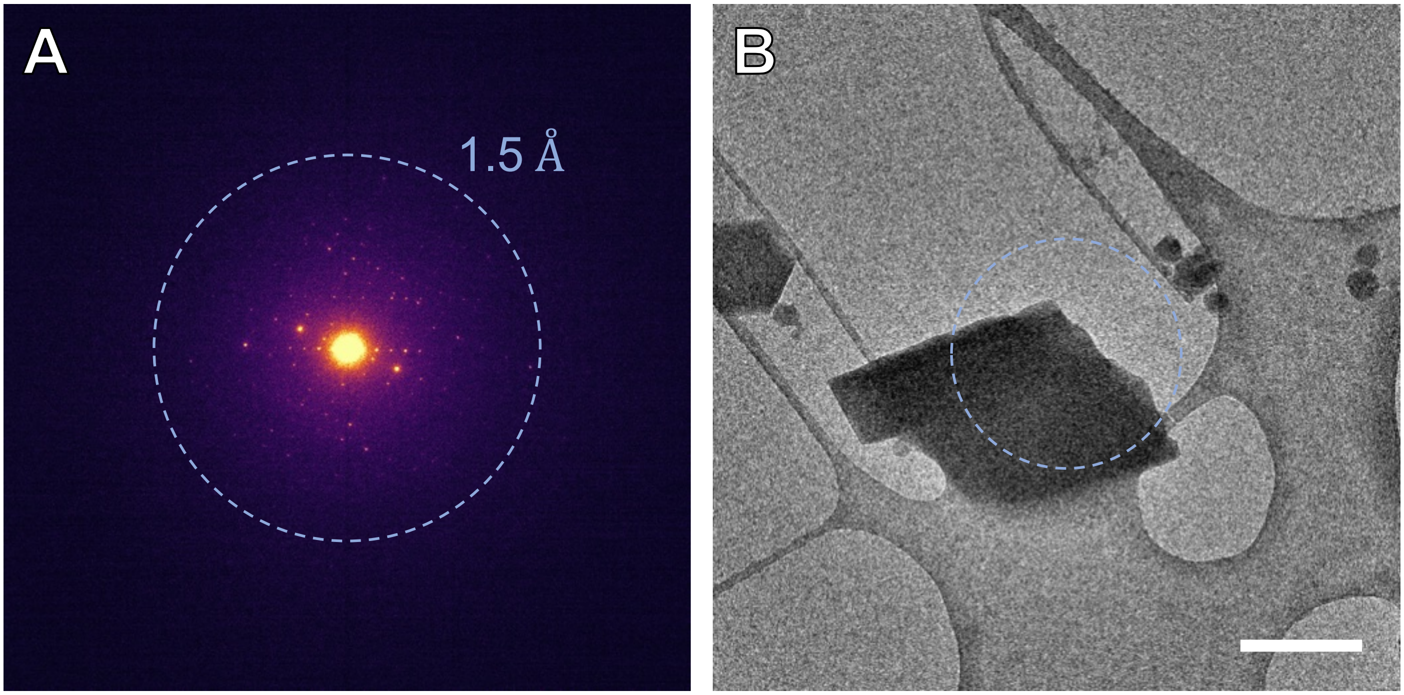

Supplementary Figure 4. Electron diffraction from fragmented block-like crystal. (**A**) Representative electron diffraction pattern (1° width) of a fragmented block-like crystal of crambin, resolution ring at 1.5 Å. (**B**) TEM image of the corresponding crystal. Dashed circle indicates beam position and diameter during diffraction. Scale bar 1 µm.

5. Supplementary Table 1. X-ray Crystallographic details for crystals of crambin.

	**Plate/needle crystal**	**Block crystal**
Temperature (K)	110	110
Crystal system	Monoclinic	Monoclinic
Space Group	$P2_1$	$P2_1$
a (Å)	22.296(4)	22.3196(2)
b (Å)	18.476(5)	18.4590(1)
c (Å)	40.84(2)	40.7640(4)
β (°)	90.64(3)	90.5399(8)
Crystal size (mm x mm x mm)	0.211 × 0.073 × 0.014	0.189 × 0.135 × 0.070
Radiation	Cu Kα (λ = 1.54184)	Cu Kα (λ = 1.54184)

6. Supplementary Table 2. Scaling and merging statistics after the STARANISO server.

	Resolution	#uniq	#Rfac	Rmerge	Rmeas	Rpim	#Isig	I/sigI	Compl. Spher.		Multiplicity		CC(1/2)	#CCAno	CC(ano)	SigAno	Compl. Ellip.	
									all	ano	all	ano					all	ano
	22.518 - 2.629	1101	41449	0.154	0.157	0.023	1101	30.595	0.9848	0.9934	37.65	21.41	0.9898	855	-0.1135	0.861	0.9848	0.9934
	2.629 - 2.071	1100	43662	0.188	0.190	0.028	1100	27.291	0.9919	0.9959	39.70	21.44	0.9834	944	-0.2474	0.932	0.9919	0.9959
	2.071 - 1.807	1103	46797	0.221	0.224	0.033	1103	24.359	0.9946	0.9970	42.43	22.61	0.9772	971	-0.1539	0.987	0.9946	0.9970
	1.807 - 1.639	1099	47977	0.287	0.291	0.043	1099	20.117	0.9928	0.9951	43.66	23.12	0.9514	981	-0.0211	0.974	0.9928	0.9951
	1.639 - 1.519	1096	49421	0.344	0.348	0.051	1096	17.650	0.9812	0.9903	45.10	23.61	0.8905	991	-0.0076	0.928	0.9812	0.9903
	1.519 - 1.426	1091	50640	0.387	0.392	0.056	1091	15.942	0.9545	0.9688	46.42	24.06	0.8896	1004	0.0230	0.898	0.9756	0.9797
	1.426 - 1.349	1077	51730	0.459	0.465	0.066	1077	13.995	0.9096	0.9200	48.03	24.88	0.8523	1000	-0.0502	0.927	0.9734	0.9727
	1.349 - 1.285	1071	53708	0.466	0.471	0.064	1071	13.567	0.8714	0.8871	50.15	25.84	0.8970	1002	-0.0212	0.878	1.0000	1.0000
	1.285 - 1.228	1088	55305	0.498	0.503	0.068	1088	12.620	0.8460	0.8609	50.83	26.13	0.9044	1030	-0.0420	0.863	1.0000	1.0000
	1.228 - 1.179	1081	55466	0.540	0.546	0.074	1081	11.486	0.8061	0.8235	51.31	26.22	0.9294	1033	-0.0672	0.802	1.0000	1.0000
	1.179 - 1.136	1099	57961	0.556	0.562	0.075	1099	11.068	0.7981	0.8140	52.74	26.92	0.9345	1057	0.1158	0.826	1.0000	1.0000
	1.136 - 1.096	1106	56867	0.645	0.651	0.088	1106	9.245	0.7697	0.7852	51.42	26.24	0.9199	1056	-0.0528	0.796	1.0000	1.0000
	1.096 - 1.062	1099	54572	0.746	0.754	0.103	1099	7.377	0.7627	0.7772	49.66	25.32	0.8983	1051	-0.0825	0.722	1.0000	1.0000
	1.062 - 1.031	1096	53665	0.895	0.904	0.125	1096	5.968	0.7400	0.7555	48.96	24.85	0.8207	1062	0.0561	0.694	1.0000	1.0000
	1.031 - 1.002	1094	50482	1.004	1.015	0.145	1094	4.862	0.7178	0.7321	46.14	23.45	0.8802	1050	-0.0237	0.638	1.0000	1.0000
	1.002 - 0.975	1091	43443	1.078	1.092	0.167	1091	4.021	0.6798	0.6936	39.82	20.18	0.8025	1057	-0.0590	0.609	1.0000	1.0000
	0.975 - 0.950	1066	40442	1.219	1.236	0.196	1066	3.256	0.6584	0.6708	37.94	19.18	0.7806	1037	-0.0157	0.594	1.0000	1.0000
	0.950 - 0.927	1034	33400	1.440	1.463	0.253	1034	2.509	0.5949	0.6081	32.30	16.34	0.6724	1001	-0.0170	0.577	1.0000	1.0000
	0.927 - 0.901	1000	23535	1.470	1.502	0.302	1000	1.904	0.4943	0.5054	23.54	11.87	0.5890	977	0.0242	0.558	1.0000	1.0000
	0.901 - 0.858	933	7978	1.416	1.502	0.482	933	1.021	0.2295	0.2213	8.60	4.55	0.3202	768	0.0427	0.513	0.8356	0.7904
Total:	22.518 - 0.858	21525	918500	0.269	0.273	0.039	21525	12.118	0.7161	0.7211	42.67	22.11	0.9922	19927	-0.1206	0.780	0.9864	0.9863

7. Supplementary Methods

a) Materials

Seeds from *Crambe abyssinica* (Meyer *Crambe*, grown near Carrington, North Dakota, 2022) were provided by the NDSU Carrington Research Extension Center. Hexanes (BDH Chemicals, >98.5%), acetone (BDH Chemicals, >99.5%), and ethanol (Pharmco, 95%, 190 proof) were used as purchased. Water was purified to 18.2 MΩ·cm using a Milli-Q system. Lacey carbon (Cu 200 mesh, Electron Microscopy Sciences) and holey carbon (Quantifoil R1.2/1.3, Cu 300 mesh) grids were used without further modifications.

b) Protein isolation and crystallization

The extraction protocol was adapted from classic literature.[55,56] A visual summary of the steps is provided in **Supplementary Figure 1.**

Seed Preparation: 230 g of seeds were manually de-husked to yield 160 g of kernels, which were ground using a mortar and pestle.

Defatting: The powder was defatted by stirring in hexanes (4 × 750 mL) for one hour per wash, with filtration after each step. The seed meal was then air-dried for 18 hours.

Acetone Extraction: The dried powder was stirred in 80% (v/v) aqueous acetone (10 mL per gram of initial seed meal) for 60 minutes and filtered. This extraction was repeated twice more.

Initial Concentration: The combined acetone filtrates were concentrated under reduced pressure (rotary evaporator, water bath at 27-30 °C) until the solution became hazy and acetone distillation ceased. The solution was stored at 4°C for 18 hours; no precipitation was observed.

Pellet Isolation: The hazy solution was centrifuged (4°C, 10,000 RPM / 16,000g, for 1hr) to yield a pellet containing a pale-yellow solid and a brown gummy layer.

Ethanolic Dissolution: The supernatant was decanted, and the pellet was fully redissolved in ~150 mL of 70% (v/v) aqueous ethanol. This solution was washed with hexanes (3 × 100 mL) to remove residual oils, with 5-8 mL of fresh ethanol added after each wash to maintain protein solubility.

Spontaneous Nanocrystal Formation: The final ethanolic solution was concentrated by half under reduced pressure (28–30 °C). While storage at 4°C did not produce crystals, we observed that when a 1 µL aliquot was placed on a glass slide, a dense field of needle-like microcrystals formed within seconds upon solvent evaporation. This crystalline slurry was used directly for MicroED grid preparation.

c) X-ray crystallography control experiments

To benchmark the quality of the spontaneously formed nanocrystals, large single crystals of two different morphologies (plates and blocks) were grown by vapor diffusion. An aliquot of the 70% ethanol solution of crambin was passed through a neutral alumina plug to remove color and then diffused against a 50% ethanol-water solution for two weeks.

Diffracted intensities were measured on a Rigaku XtaLAB Synergy-S diffractometer with a HyPix-6000HE detector and a Cu-target PhotonJet-S microfocus source (λ = 1.54184 Å). Crystals were cryo-protected in a 1:1 mixture of mother liquor and glycerol and maintained at 110 K in a nitrogen cryostream. Data were collected, indexed, integrated, and scaled using CrysAlisPro.[59] A single 14 µm needle, representative of the morphology used for MicroED, was tested with X-rays. It diffracted poorly to only 1.6 Å and showed signs of rapid radiation damage, rendering it unsuitable for X-ray structure determination. These were the crystals subsequently crushed for the comparative MicroED experiments. In contrast, the block-like crystals grown by vapor diffusion and later crushed for MicroED experiments diffracted to beyond 0.8 Å resolution on this X-ray diffractometer. The unit cell parameters are reported in **Supplementary Table 1**.

d) MicroED grid preparation

Holey-carbon (Quantifoil Cu 300 R1.2/1.3) or lacey carbon (Cu 200, EMS) grids were glow-discharged at 15 mA for 30s using a Pelco Easi-Glow. Grids were transferred to either a Leica GP2 or a Thermo Fisher Vitrobot Mark IV plunge freezer. The GP2 was equilibrated to 4 °C and 95% relative humidity and the Vitrobot was set to 22°C and 100% relative humidity for 15-20 minutes prior to use. After allowing the grid to equilibrate in the chamber for 30s, a 3 µL aliquot of the crystalline slurry was applied. Grids were blotted manually from the back side for approximately 10s inside of the Vitrobot and for 30s from behind automatically in the GP2 before being plunge-frozen in liquid ethane.

e) TEM lattice imaging

High resolution TEM images of crystal lattices were collected at a nominal magnification of 120kx corresponding to a pixel size of 0.851 Å using a Thermo Fisher Glacios operating at 200kV at liquid nitrogen temperature. A Falcon 4 direct detector operating in counting mode was used to save the data in EER format with an exposure time of 3.48s corresponding to 812 EER frames. The total exposure was ~37 $e^{-}/Å^2$. MotionCor2[60] version 1.6.4 was used to align the movie frames using 116 fractions, 1x EER upsampling, and no dose weighting applied.

f) MicroED data collection

MicroED data were recorded on a Thermo Fisher Glacios transmission electron microscope operated at 200 kV (λ = 0.0251 Å) at liquid nitrogen temperatures. A Falcon 4 direct electron detector was used for data acquisition under the control of SerialEM.[57,58] The microscope was set to microprobe mode with a 20 μm C2 aperture and no SA aperture, resulting in a parallel beam of approximately 2 μm diameter. A two-part strategy was employed to maximize the dynamic range: half of the datasets were collected in linear mode and half in counting mode. For linear-mode wedges, the total exposure was ~1.6 $e^{-}/Å^2$ over a 60s collection with continuous stage rotation, resulting in a tilt of 0.20° per 1-second frame. For counting-mode wedges, the total exposure was ~0.8 $e^{-}/Å^2$, with a finer slicing of 0.10° per 1-second frame. The total accumulated dose per crystal was kept below 2.0 $e^{-}/Å^2$. Starting angles of -60°, -30°, and 0° were used for collection of 60° linear-mode wedges. Counting-mode 30° wedges were collected with starting angle varying from -60° to +30° in 15° increments.

g) Conversion of MRC movies to miniCBF format

Raw movie stacks in MRC format were converted to the miniCBF format suitable for crystallographic processing using a custom Python script, which leverages the FabIO,[61] NumPy,[62] and SciPy[63] libraries. This pipeline performed several critical pre-processing steps.

Metadata Extraction: For each movie, experimental parameters were parsed from the corresponding .mdoc file. These included the unbinned camera pixel size (14.0 μm), accelerating voltage (200 kV), camera length (961.06 mm), and frame-by-frame goniometer rotation angles.

Binning: The raw 4096 x 4096 (or 2048 × 2048 if hardware binned) pixel frames were binned by a factor of 4 (or 2) in both X and Y dimensions, producing final 1024 × 1024 pixel images with an effective pixel size of 56 μm.

Pedestal Correction: To ensure all pixel counts were positive in the linear mode datasets, a pedestal value was automatically calculated by finding the minimum integer value in the entire image stack (e.g., -159 for one dataset) and adding its absolute value to every pixel.

Beam Center Correction: A two-pass algorithm was applied to correct for beam drift during data collection. In the First Pass / Peak Finding step, an initial beam center was located by applying a Gaussian blur (σ=3.0) to a 100×100 pixel central region of interest and fitting a 2D Gaussian function to the resulting peak for each frame in the movie. The second pass consists of Smoothing and Validation. Any center that jumped more than 2.0 pixels from its predecessor was marked as an outlier. A Savitzky-Golay smoothing filter (window length=11, polynomial order=2) was then applied to the path of the valid centers. Outliers and missing centers were filled in using the smoothed path. Finally, each frame was shifted to a common, smoothed center using linear interpolation before being written to a miniCBF file with a complete header.

h) Data integration, scaling, and anisotropy correction

Corrected CBF files were processed with XDS[64] (BUILD 20230630). Datasets were initially indexed in space group *P* 1, *P* 2 or *P* 2 2 2. The resulting INTEGRATE.HKL files were analyzed with POINTLESS (CCP4 9.0) to determine the correct lattice symmetry, which was consistently identified as monoclinic, space group $P2_1$. Each dataset was then re-integrated in XDS using the correct symmetry and a fixed reference unit cell.

From an initial pool of over 77 crystals that showed diffraction, a final selection of 58 datasets that showed high mutual correlation coefficients and consistent cell parameters were scaled together using XSCALE[65] after clustering and pruning down using XSCALE_ISOCLUSTER (KD 2024-10-27). The final scaling process used a target unit cell of a = 41.49 Å, b = 18.79 Å, c = 22.52 Å, β = 90.84°- the median of the cell values used in the scaling and merging steps. The resulting merged reflection file, XSCALE.1.HKL, contained 937,603 total observations of 26,147 unique reflections and was used as the input for anisotropy analysis using the STARANISO[29] server. The full scaling and merging statistics for the final dataset are reported in **Supplementary Table 2.**

i) Phasing, Model Building, and Refinement

Ab initio Phasing and Automated Building: Phasing was performed using a multi-step procedure. First, an ideal 5-residue α-helix (theor-helix-5.pdb from the CCP4[66] fragment library) was placed using molecular replacement in PHASER[33] (via the Phenix command-line utility). The best solution yielded

an LLG of 64 and a TFZ of 7.2. The phases from this solution were used to initiate density modification in ACORN.[34] ACORN was run with default parameters, terminating after 48 cycles and reaching a map correlation of 68.9%. The resulting map was used as input for BUCCANEER,[37] which built the complete model in one pass. Based on the clarity of the density modified and refined density maps in regions of known microheterogeneity (residues 22 and 25),[38] the model was built and refined using both potential side chains which appeared clearly in the difference densities and the occupancies were allowed to refine, resulting in an approximately 50/50 mixture of the PL and SI isoforms.

Crystallographic Refinement: The final model was refined in REFMAC5[67,68] (v5.8.0430) using electron scattering factors. The protocol involved 5 cycles of manual model adjustment in Coot followed by automated refinement. Psuedo-merohedral twinning[69] was modeled using the TWIN -h -k l instruction with a refined twin fraction of 10.0%. Anisotropic B-factors and riding hydrogen atoms were included in the final rounds. Final model statistics and validation metrics from MolProbity[70] are reported in **Table 1.**

j) Figure generation.

Plots were generated using Python 3.11. Diffraction patterns and sums were produced using FIJI.[71] Figures were arranged using PowerPoint. Density figures and models were prepared using UCSF ChimeraX[72] 1.9 and PyMol[73] 3.1.

Supplementary Video 1. The movie shows the placement of the 5-alanine fragment on the white cartoon of the final structure of crambin. The $2F_o$-F_c map of the phases from this initial placement are in blue surfaces at a 1σ contour around the protein with a range of 3 Å.

Supplementary Video 2. The movie shows the placement of the 5-alanine fragment on the white cartoon and sticks of the final structure of crambin. The |E| map directly after density modification from this initial fragment in ACORN are in orange surfaces at a 1.5 σ contour around the protein with a range of 3 Å, showing clear, individual spherical densities around individual atoms.
